# Predictors of adherence to antiretroviral therapy among people living with HIV and AIDS at the regional hospital of Sokodé, Togo

**DOI:** 10.1186/1471-2458-14-1308

**Published:** 2014-12-19

**Authors:** Issifou Yaya, Dadja Essoya Landoh, Bayaki Saka, P’Niwè Massoubayo Patchali, Peter Wasswa, Abdoul-samadou Aboubakari, Mathias Kouamé N’Dri, Akouda Akessiwe Patassi, Koussake Kombaté, Palokinam Pitche

**Affiliations:** Laboratoire de Santé Publique (EA 3279), Aix-Marseille Université, Marseille, France; Division de l’Epidémiologie, Ministère de la Santé. S/C INH - Togo, BP 1396, Lomé, Togo; Service de dermatologie et IST, CHU Sylvanus Olympio, Université de Lomé, Lomé, Togo; Centre Hospitalier Régional (CHR) de Sokodé, Service de dispensation d’antirétroviraux (ARV), Sokodé, Togo; African Field Epidemiology Network (AFENET), Kampala, Uganda; Service de gynéco-obstétrique, CHU- Kara, Kara, Togo; Service des Maladies Infectieuses et de Pneumologie, CHU Sylvanus Olympio, Lomé, Togo; Conseil National de Lutte contre les IST/VIH/Sida, Lomé, Togo

**Keywords:** Adherence to ART, People Living With HIV/AIDS, Togo

## Abstract

**Background:**

Adherence to antiretroviral therapy (ART) is beneficial in reducing the risk of emergence of HIV resistant strains. Adherence to ART among Persons Living with HIV/AIDS (PLWHA) is influenced by several factors related to the patient, the medication, and health facilities. In Togo, previous studies on adherence to ART have reported good adherence to ART during the first year of follow-up. However these may hide many disparities dues to cultural specificities which may differ across geographic areas of the country. We sought to determine the level of adherence to ART and document the associated factors among PLWHA at the regional hospital of Sokodé, Togo.

**Methods:**

This was an analytical cross-sectional study conducted from May to July 2013 at the regional hospital of Sokodé among 291 PLWHA who had been on ART for at least three months before the study.

**Results:**

A total of 291 PLWHA on ART were enrolled in the study. The mean age (±SD) was 37.3 ± 9.3 years and the sex ratio (Male/Female) was 0.4. Among them, 195 (67.0%) were living with their partners and 210 (72.2%) had formal education. Two-thirds (194/291; 66.7%) of the PLWHA interviewed lived in urban areas. The global adherence to ART was 78.4%; the factors associated with ART adherence were: level of education (aOR = 3.54; *p = 0.027*), alcohol consumption (aOR = 0.43; *p = 0.033*), ART perception (aOR = 2.90; *p = 0.026*) and HIV status disclosure to sexual partner (aOR = 7.19; *p ≤ 0.001*).

**Conclusion:**

Although the level of adherence to ART in this study was higher than those reported in some studies in Sub-Saharan Africa, it remains sub-optimal and needs improvement. This may therefore hinder the implementation of efficient interventions related to access to ART services.

## Background

The number of people living with HIV and AIDS (PLWHA) was estimated at 23.5 million in Sub-Saharan Africa, where 1.2 million of deaths related to AIDS had been recorded in 2012 [1]. The “3 by 5” and “Treatment 2015” an initiative launched jointly by WHO and UNAIDS in 2003 and 2011 respectively have created a truly dynamic environment in HIV care for PLWHA in developing countries. Access to antiretroviral treatment (ART) has considerably expanded since then, to reaching 9.7 million persons in resource-limited countries in late 2012 [2]. This has led to the reduction of not only the number of new HIV infections, but also in AIDS mortality and -its related co-morbidities. The introduction of ART in the treatment of PLWHA has dramatically changed the course of HIV/AIDS epidemic, making AIDS a chronic disease. Thus, new issues related to the chronicity of the disease have appeared, including the adherence to ART [3].

Indeed, adherence to ART is essential for long-term therapeutic success and is therefore a major concern to ART programs. Studies have shown that good adherence to ART has huge benefits; because it is necessary not only in reducing the risk of emergence of HIV resistance strains, but also in improving the health status of the PLWHA [4, 5]. To date, research on ART adherence includes methodologically rigorous investigations; however the measurement of adherence to ART raises many questions because there is no standardized measurement tool which may be appropriate in all cases. In low-income countries, several studies have been conducted among PLWHA on ART in order to measure their adherence to ART [4–14]. The methods used were either subjective methods such as self-reporting questionnaires [7, 8], or objective methods such as counting the remaining tablets, or often the combination of these two methods [6]. Adherence to ART in these studies was estimated at 55.4% in Congo [6] and 93% in Senegal [9]. It is also well documented that adherence to ART among PLWHA is influenced by several factors related to the patient, the medication, the psychosocial environment and the medical care facilities [15, 16].

In Togo, programs against HIV/AIDS introduced ART in the late 90s, and the number of PLWHA on ART increased from 6,721 in 2006 to 30,334 in 2012, with an increase of ART coverage from 20% in 2006 to 49.3% in 2012 [17]. Previous nationwide studies on adherence to ART in Togo had reported rates of adherence during the first year of follow-up, estimated at 89.6% [18], in 2009 and 78% in 2011 [17]. However these rates hide many geographical disparities dues to cultural specificities which may differ across geopolitical zones of the country. The assessment of the adherence to ART could be an opportunity to identify potential obstacles to taking medication in order to prevent treatment failure and the development of drug resistance among PLWHA. In addition, published studies about factors that influence adherence to ART in Togo are limited. We sought to determine the level of adherence to ART and to document the factors associated with ART adherence among PLWHA at the regional hospital of Sokodé in Togo.

## Methods

### Study design

This was a cross-sectional study conducted at regional hospital of Sodoké over a period of three months from May to July 2013 among PLWHA on ART for at least three months.

### Setting

The regional hospital of Sokodé is a health reference center of the central region which is one of the six health regions of Togo. The regional hospital of Sokodé is located about 350 km from the capital Lomé. It serves four health districts with a total population of 654,074 inhabitants in 2013 [19]. Around 45% of the 1,869 PLWHA registered in the central region are followed up in this hospital [17].

### Study population and sampling (Figure 1)

This study targeted PLWHA who were under ART and followed up in the regional hospital of Sokodé.

**Figure 1:**
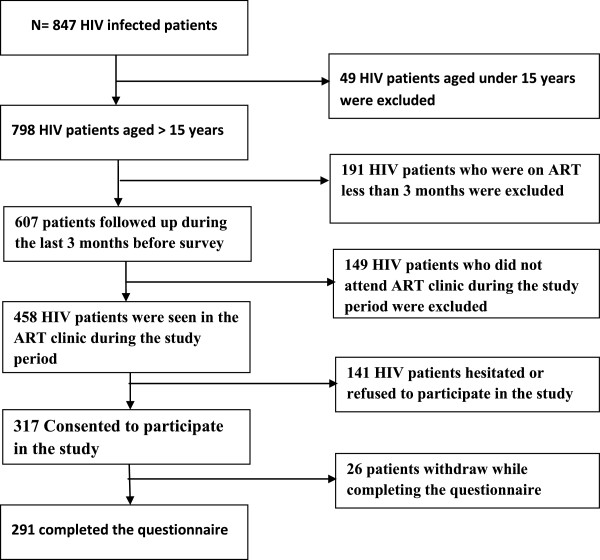
**Flow chart of study sampling of PLWHA on ART in Sokodé, Togo.**

Of the 843 PLWHA who were followed in the regional hospital of Sokode, 798 were at least 15 years old [17].

We used a purposive and comprehensive sampling to include PLWHA aged 15 years or older, who were taking ART for at least three months and were followed up in the regional hospital of Sokodé [20]. All patients attending the HIV care clinic for follow up during the study period and who met the inclusion criteria were invited to participate in the study.

HIV positive pregnant women were not included in the survey because of the difference of ART regimen for this target group.

### Data collection

Data were collected through a questionnaire-assisted interview in French or in the local language, conducted by the clinic officers to ensure good understanding of the questions.

The questionnaire included socio-demographic information, clinical features, information on the adherence to ART and information on HIV/AIDS knowledge. Data on HIV status disclosure to the sexual partner and sexual behavior aspects were collected only among sexually active PLWHA. Sexual activity was defined as reporting at least one sexual partner during the previous 3 months [20].

To quantify the level of adherence in our study, three measurements tools were used, including: i) self-reportingof missed doses: During interview of the patient for a possible missed medication during the last four days; the patient was considered adherent if he had not missed to take any medication during the previous four days. ii) Counting the remaining tablets: The caregiver records the number of tablets or capsules remaining and evaluates adherence by considering a missed tablet or capsule as a tablet or capsule absorbed. In Togo, PLWHA on ART are requested to renew their treatment every month during the follow up appointment at the HIV care clinics at least three days before the end of their previous prescription. Patients with a percentage of intake tablets or capsules equal to or greater than 95% were considered adherent. iii) the rate of prescriptions renewed, here expressed as the number of appointments honored during the three months preceding the survey. Patients who did not fail any appointment were classified as adherent.

The global index of adherence was obtained by summing the results of the different measurement of adherence methods used in the study (a coefficient assigned to each item with 0 for non adherence and 1 for adherence). Thus the global index of adherence was used to classify PLWHA in two categories according to the level of adherence to ART: poor adherence (index from 0 to 1), good adherence (index from 2 to 3).

### Data analysis

Data entry was performed using EpiData software version 3.1 and analyzed in SPSS Inc version 17.0 (SPSS Inc, Chicago, IL, USA).

For continuous variables, mean, median and standard deviation were calculated while for categorical variables we calculated proportions. Our primary outcome of interest was people living with HIV who were adherent to ART compared to PLWHA who were non-adherent. Pearson chi-square test or Fisher′s exact test were used when appropriate in bivariate analysis.

Multivariate backwards stepwise logistic regression analysis was performed to identify independent risk factors for the primary of being adherent or non-adherent to ART. All variables significant during bivariate analysis at a p-value less than 0.05 were introduced in a logistic regression model to appreciate the adjusted effect and derive the adjusted odds ratio (aOR) of each on the primary outcome, "global adherence to ART" expressed as a dichotomous variable. A 95% level of confidence was applied throughout.

### Ethical issues

This study was approved by the National AIDS and STI Program of Togo (Ref N° 098/2013/MS/DSSP/PNLS-IST). We obtained consent from patients that participated in the study. For each person interviewed, the objectives, benefits to participate in the survey and progress of the investigation were clearly stated as well as their right to interrupt the interview without justification. An informed consent form was signed after the verbal explanation was made by the investigating officer in the language understood by the participant. The survey was anonymous and confidential. The respondents’ names were not recorded to ensure their confidentiality; these were replaced by numbers.

## Results

### Characteristics of the study population

Of the 458 PLWHA on ART who were seen in the hospital, 317 consented to participate in the study. Of these, 291 PLWHA completed the interview including 201 (69.1%) women. The mean age of the participants was 37.3 ± 9.3 years (range: 18–68 years). Among the 291 PLWHA, 195 (67.0%) were living with their partners and 210 (72.2%) had at least primary level of education. Two-thirds (194/291; 66.7%) of the PLWHA interviewed lived in urban areas.

On clinical and therapeutic level, 106 (36.4%) of the PLWHA were at WHO clinical stage III and IV at the time of the survey. The median duration of ART among PLWHA was 35 months. At the last check-up prior to the survey, the average of CD4 count was 381 ± 209cells/mm^3^ (range 9 to 1080 cells/mm^3^). Most PLWHA were on first-line treatment (274/291; 94.2%) while 83.5% (243/291) were on Zidovudine/Lamivudine/Nevirapine therapeutic combination. At the time of the survey, 217 (74.6%) of the PLWHA interviewed were sexually active since initiating ART and of these, 60.9% (131/217) had disclosed their HIV status to their sexual partner. In addition, 130 PLWHA (60.5%) knew their sexual partner’s HIV status (Table 1).

**Table 1:** **Descriptive data of PLWHA on ART at the regional hospital of Sokodé, Togo**

Patients characteristics	N (%)	Measurement of adherence			Global adherencen (%)	p-value
		***Self-report***	***Appointment***	***pills count***		
		***n (%)***	***n (%)***	***n (%)***		
**Sex**		*	°	°		0.283
*Male*	*90 (30.9)*	73 (81.1)	78 (86.7)	74 (82.2)	74 (82.2)	
*Female*	*201 (69.1)*	139 (69.2)	172 (85.6)	144 (71.6)	154 (76.6)	
**Age**		°	°	*		0.168
*Less than 25 years*	*26 (8.9)*	21 (80.8)	25 (96.2)	17 (65.4]	21 (80.8)	
*25-35 years*	*109 (37.5)*	74 (67.9)	91 (83.5)	75 (68.8)	79 (72.5)	
*More than 35 years*	*156 (53.6)*	117 (75.0)	134 (85.9)	126 (80.8)	128 (82.1)	
**Marital status**		°	°	°		0.330
*Single/widower/divorced*	*96 (33.0)*	64 (66.7)	84 (87.5)	68 (70.8)	72 (75.0)	
*Married/in couple*	*195 (67.0)*	148 (75.9)	166 (85.1)	150 (76.9)	156 (80.0)	
**Education level**		**	*	**		**0.008**
*Non schooled*	*81 (27.8)*	61 (75.3)	73 (90.1)	59 (72.8)	67 (82.7)	
*Primary school*	*105 (36.1)*	62 (59.0)	83 (79.0)	69 (65.7)	71 (67.6)	
*Secondary school*	*84 (28.9)*	72 (85.7)	77 (91.7)	71 (84.5)	73 (86.9)	
*High level*school	*21 (7.2)*	17 (81.0)	17 (81.0)	19 (90.5)	17 (81.0)	
**Religion**						0.287
*None or traditional*	*15 (5.2)*	11 (73.3)	13 (86.7)	11 (73.3)	11 (73.3)	
*Islam*	*149 (51.2)*	103 (69.1)	126 (84.6)	108 (72.5)	112 (75.2)	
*Christian*	*127 (43.6)*	98 (77.2)	111 (87.4)	99 (78.0)	105 (82.7)	
**Place of residence**		*	°	°		0.131
*Urban*	*194 (66.7)*	133 (68.6)	165 (85.1)	145 (74.7)	147 (75.8)	
*Rural*	*97 (33.3)*	79 (81.4)	85 (87.6)	17 (75.3)	81 (83.5)	
**Alcohol consumption**		**	*	°		≤**0.001**
*No*	*223 (76.6)*	173 (77.6)	197 (88.3)	173 (77.6)	187 (83.9)	
*Yes*	*68 (23.4)*	39 (57.4)	53 (77.9)	45 (66.2)	41 (60.3)	
**Clinical WHO stage**		°	°	°		0.242
*Stage I*	*53 (18.2)*	39 (73.6)	45 (84.5)	39 (73.6)	41 (77.4)	
*Stage II*	*132 (45.4)*	95 (72.0)	113 (85.6)	101 (76.5)	109 (82.6)	
*Stage III and IV*	*106 (36.4)*	78 (73.6)	92 (86.8)	78 (73.6)	78 (73.6)	
**Duration of ART therapy**		°	*	°		0.379
*Less than one year*	*33 (11.3)*	28 (84.8)	32 (97.0)	26 (78.8)	28 (84.8)	
*1 to3 years*	*126 (43.3)*	94 (74.6)	113 (89.7)	95 (75.4)	101 (80.2)	
*More than 3years*	*132 (45.4)*	90 (68.2)	105 (79.5)	97 (73.5)	99 (75.0)	
**CD4’s count (last check-up)**		*	*	°		**0.022**
*CD4 ≤ 350*	*116 (39.9)*	76 (65.5)	93 (80.2)	83 (71.6)	83 (71.6)	
*CD4 > 350*	*175 (60.1)*	136 (77.7)	157 (89.7)	135 (77.1)	145 (82.9)	
**Therapeutic’s line**		°	°	°		0.574
*1* ^*st*^ *line*	*274 (94.2)*	200 (73.0)	236 (86.1)	204 (74.5)	216 (78.8)	
*2* ^*nd*^ *line*	*12 (4.1)*	9 (75.0)	11 (91.7)	9 (75.0)	9 (75.0)	
*3rd line*	*5 (1.7)*	3 (60.0)	3 (60.0)	5 (100.0)	3 (60.0)	
**Type of ART prescribed**		°	°	°		0.310
*Triomune*	*3 (1.0)*	3 (100.0)	3 (100.0)	3 (100.0)	3 (100.0)	
*Duovir/EFV*	*36 (12.4)*	22 (61.1)	31 (86.1)	25 (69.4)	27 (75.0)	
*Duovir/NVP*	*243 (83.5)*	181 (74.5)	210 (86.4)	182 (74.9)	192 (79.0)	
*TNF/3TC/Aluvia*	*7 (2.4)*	4 (57.1)	4 (57.1)	6 (85.7)	4 (57.1)	
*ABC/DDI/Aluvia*	*2 (0.7)*	2 (100.0)	2 (100.0)	2 (100.0)	2 (100.0)	
**Reported side effects**		°	°	°		0.988
*No*	*240 (82.5)*	175 (72.9)	210 (87.5)	178 (74.2)	188 (78.3)	
*Yes*	*51 (17.5)*	37 (72.5)	40 (78.4)	40 (78.4)	40 (78.4)	
**ART perception by patient**		**	**	**		≤**0.001**
*Low*	*33 (11.3)*	*15 (45.5)*	*21 (63.6)*	*15 (45.5)*	*15 (45.5)*	
*Good*	*258 (88.7)*	*197 (76.4)*	*229 (88.8)*	*203 (78.7)*	213 (82.6)	
**HIV-status disclosure to sexual partner**		**	**	**		≤**0.001**
*No*	*84 (39.1)*	39 (46.4)	62 (73.8)	48 (57.1)	46 (54.8)	
*Yes*	*131 (60.9)*	106 (80.9)	119 (90.8)	109 (83.2)	113 (86.3)	
**Knowledge of the HIV sero-status of the sexual partner**		**	*	**		≤**0.001**
*No*	*85 (39.5)*	43 (50.6)	65 (76.5)	49 (57.6)	51 (60.0)	
*Yes*	*130 (60.5)*	102 (78.5)	116 (89.2)	108 (83.1)	108 (83.1)	
**Self-report adherence to ART**						
*No*	*79 (27.1)*					
*Yes*	*212 (72.9)*					
**Appointment’s adherence to ART**						
*No*	*41 (14.1)*					
*Yes*	*250 (85.9)*					
**Pills count’s adherence to ART**						
*No*	*73 (25.1)*					
*Yes*	*218 (74.9)*					
**Global adherence to ART**						
*No*	*63 (21.6)*					
*Yes*	*228 (78.4)*					

° : p > 0.05; * : p < 0.05; ** : p < 0.001.

### ART adherence

Out of the 291 PLWHA who responded to the questionnaire, 212 (72.9%) had not skipped any tablet within four days prior to the survey. Through this tool of measure, the proportion of adherent was significantly higher among men (81.1%; *p <0.05*); PLWHA in rural areas (81.4%; *p < 0.05*) and among patients who had secondary education (85.7%; *p < 0.001*). Adherence to ART was also significantly associated with alcohol consumption (*p < 0.001*), CD4 count (*p < 0.05*), ART perception (*p < 0.001*), HIV-status disclosure to sexual partner (*p < 0.001*) and knowledge of the HIV sero-status of the sexual partner (*p < 0.001*) (Table 1).

Most of patients 250 (85.9%) had complied with the appointment renewal of ART prescriptions. The proportion of PLWHA who had respected appointments for ART renewal decreased with the duration of being under ART (*p < 0.05*). But this proportion was high among patients who had secondary education (91.7%; *p < 0.05*), among those who did not consume alcohol (88.3%; *p < 0.05*) and among patients who had a good perception of ART (88.8%; *p < 0.001*). In addition, the number of CD4 (*p < 0.05*), HIV-status disclosure to sexual partner (*p < 0.001*), knowledge of the HIV sero-status of the sexual partner (*p < 0.001*) were also associated with the adherence to ART according to this method (Table 1).

On counting the remaining tablets, 218 PLWHA (74.9%) had taken 95% or more of their ART. According to this tool, adherence to ART increased significantly with the age of the PLWHA (*p < 0.05*). This proportion was high among patients who had high school education (90.5%; *p < 0.001*), and among patients who had a good perception of ART (78.7%; *p < 0.001*). With this tool, the adherence was also significantly associated with HIV-status disclosure to sexual partner (*p < 0.001*), knowledge of the HIV sero-status of the sexual partner (*p < 0.001*) (Table 1).

Among the PLWHA of regional hospital of Sokodé, the global adherence to ART was calculated to be 78.4% (228/291). In bivariate analysis, the global adherence to ART was statistically associated with the level of education (*p = 0.008*), alcohol consumption (*p ≤ 0.001*), ART perception (*p ≤ 0.001*), knowledge of HIV status of the sexual partner (*p ≤ 0.001*), HIV status disclosure to the sexual partner (*p ≤ 0.001*) and CD4 count at last check-up (*p = 0.022*) (Table 1).

In multivariate analysis, four factors remained significantly associated with good global adherence to ART: PLWHA with a secondary education were about four times more likely to have a good adherence to ART (*p = 0.022*). Also, those who consumed alcohol daily were significantly less likely to be adherent to ART (*p = 0.041*). Patients who had a good perception of ART were almost three times more likely to have good adherence to ART compared to those who had poor perception (*p = 0.026*). Finally, PLWHA who disclosed their HIV status to their sexual partners (*p = 0.000*) were seven times more likely to have good adherence to ART (Table 2). The knowledge of HIV status of the sexual partner and the CD4’s count at last check-up which were significant in bivariate analysis were not significant in multivariate analysis.

**Table 2:** **Predictor factors of adherence of PLWHA to ART at the regional hospital of Sokodé, Togo**

Patients characteristics	aOR	95% for a OR	p-value
**Education level**			
*Non schooled*	Ref		
*Primary school*	0.77	*[0.31 ; 1.92]*	0.573
*Secondary school*	3.54	*[1.16 ; 10.81]*	**0.027**
*High level*school	1.16	*[0.28 ; 4.84]*	0.837
**Alcohol consumption**			
*No*	Ref		
*Yes*	0.43	*[0.20 ; 0.93]*	**0.033**
**ART perception**			
*Low*	Ref		
*Good*	2.90	*[1.14 ; 7.35]*	**0.026**
**Knowledge of the HIV sero-status of the sexual partner**			
*No*	Ref		
*Yes*	0.48	*[0.17 ; 1.50]*	0.209
**HIV-status disclosure**			
*No*	Ref		
*Yes*	7.19	*[2.38 ; 21.68]*	≤**0.001**
**CD4’s count (last check-up)**			
*CD4 ≤ 350*	Ref		
*CD4 > 350*	1.87	*[0.90 ; 3.88]*	0.092

## Discussion

This study noted that four factors were associated with the global adherence to ART among PLWHA on ART attending the regional hospital of Sokodé. These were: level of education, alcohol consumption, ART perception and HIV status disclosure to sexual partner.

In fact we used three different tools in order to assess the global adherence among the HIV-patient included in the study. Firstly with the self-reporting tool, 72.9% of the patients had a good adherence. Lower adherence rate with this tool had been reported, 62.8% [4] in a study conducted in Nigeria. The self-reporting method is the most common tool used to access the adherence to ART among HIV-patients [6–14]. Even if it is a subjective tool to measure adherence to ART, we had used it in a short period to minimize a recall bias which could occur. With the second methods using pills count’s tool, 74.9% PLWHA had good adherence to ART. Finally the number of appointments honored during the three months preceding the survey showed that 85.9% of the PLWHA were adherent. If this tool seems to have overestimated the adherence to ART in our study, it remains a reliable and reproducible method. Overall, the only advantage of using these methods is that they are at low cost and easy to implement [21]. The combination of these methods provides more consistent and reliable results [14].

In our study, 78.4% of the PLWHA on ART globally adhered to this treatment according to the three methods. This adherence rate is sub-optimal (defined as less than 95%), but it is higher than that reported by Potchoo *et al.* (62.6%) in Lomé and Sokodé [22]. Many studies have reported that a PLWHA requires a minimum level of adherence to ART of 95% in order to prevent development of drug resistance and disease progression [8, 23]. In fact, an adherence to ART rate of 95% or greater is essential to obtain a decrease of the viral load and morbidity related to HIV infection. We found that 21.6% of the patients were non-adherent to ART. This suggests all the difficulties encountered by health personnel in health facilities to retain PLWHA on ART to achieve good adherence to treatment.

This finding is consistent with that found in a meta-analysis which reported that 77% of patients in Africa achieved adequate adherence. It is also similar to the ART adherence rate reported in India (74%) [13], in southern Ethiopia (74.4%) [24], but lower than ART adherence rate reported in studies conducted in Kenya (82%) [12], in Nepal (85.5%) [25] in West Africa (91.8%) [26] and in Senegal (93.0%) [9]. However, this disparity of adherence rates across studies may depend on the context and the methods of measuring adherence which may vary from a study to another.

Our study shows that PLWHA who attended at least secondary level of school were almost 3.6 more likely to adhere to ART. Indeed, formal education obviously plays a major role in understanding and communicating information related to health care. Educated people may show better adherence to ART due to their ability to follow the instructions related to the treatment given by the health providers. Other studies [4, 25] supported that ART adherence rate was higher among HIV-infected patients who had a high level of formal education. Our finding was also consistent with that reported by Kalichman *et al.* [27] in United States of America, who had earlier identified low educational status as a major factor of non-adherence to ART. Lower education was found to have associations with barriers to receiving HIV related health care, including ART. But this result is not consistent with those reported by some other studies in developing countries [15, 28] which found that the majority of patients who had low adherence to ART were literate. In these studies, authors argued that situation may be due to the fact that these categories of PLWHA had busy occupations that hindered taking antiretroviral drugs.

Secondly, the consumption of alcohol was a poor predicator of ART adherence. Alcohol consumption negatively affects HIV-infected patients by reducing their adherence to ART about 40% to 50% [29]. In fact alcohol intake increases forgetfulness by patients of the times and instructions of ART medication. This finding is well documented in prior studies [14, 25, 28, 30]. In a meta-analysis of alcohol consumption among PLWHA [28], adherence to ART was found low among individuals who usually drank alcohol. Jaquet *et al.* [26] also reported that alcohol consumption and hazardous drinking were significantly associated with non-adherence to ART among HIV-infected patients in West Africa. In a study conducted in Botswana [31], about 40% of the participants reported that they had missed a dose of ART due to alcohol consumption. The implication of this finding is mainly to implement a strategic intervention toward HIV-infected patients who have an alcohol use background in order to provide them appropriate health care, psychological support and improve ART outcomes.

In our study, PLWHA who had good perception of ART, were almost three times more likely to adhere to ART. The level of belief in the benefits and effectiveness of ART could also promote adherence as documented by several studies [24, 32, 33]. This was particularly found in patients who have experienced serious events related to HIV infection and whose state of health has been improved after ART initiation. In a cross-sectional mixed-methods study carried out in Nepal, Wasti *et al.* [25] found that negative perceptions could act as barriers to adherence to ART. Our finding underscores the relevance of health education during the patients’ visits which could help them to overcome negative experiences of the side effects of ART.

Finally, those who disclosed their HIV sero-status to the sexual partner were nearly seven times more likely to adhere to ART. Several studies have shown that HIV sero-status disclosure is a known predictor of increased adherence to ART [4, 10, 11, 25, 30, 34]. In fact, HIV disclosure could be the first stage of creating a supportive relationship with the sexual partner and with the family, and that would facilitate the acceptance and the continuation of ART. Literature suggests that HIV disclosure facilitates ART adherence [11, 34]. In a study conducted in Ibadan, Nigeria, Olowookere *et al.* [10] reported that, compared to the HIV positive patients who disclosed their HIV status, those who were unwilling to disclose their HIV status were more likely to be non-adherent to ART. In a meta-analysis of adherence to ART in Sub-Saharan Africa and North America, Mills *et al.* [30] found that PLWHA who did not disclose their HIV status were more likely to report frequent ART interruptions and fear to be discriminated or stigmatized. Health care providers and the policy makers need to convince HIV-infected patients who are still reluctant to disclose their HIV status to do so. These interventions should include social institutions like nongovernmental organizations, HIV-infected persons associations and confessional institutions which play an important role in facilitating treatment, and promoting adherence to ART.

### Limitations of study

The findings of this study should be interpreted in view of some limitations. This study was conducted among PLWHA followed up only at the regional hospital of CHR Sodoké. Therefore, the sample may not be representative of the whole country. Due to the cross-sectional study design of this study, the variation of adherence of the participants to ART which can occur over the time was not assessed.

## Conclusion

The level of adherence to ART was sub-optimal at the regional hospital of Sokodé. The level of education, alcohol consumption, ART perception, and HIV sero-status disclosure to the sexual partner were the main factors associated with the adherence to ART. Policy-makers, NGOs and health care providers should give priority to relevant interventions that focus on regular follow-up, education of the PLWHA, the negative consequences of alcohol consumption on ART adherence, and provide a supportive environment that encourages HIV-infected patients to disclose their HIV sero-status in confidence.

## Abbreviations

aOR: adjusted odds ratio
ART: Antiretroviral therapy
PLWHA: People Living With HIV and AIDS.
